# Supplementary material for: MorphoNet: an interactive online morphological browser to explore complex multi-scale data
Source: Nat Commun. 2019 Jun 27;10:2812. doi: 10.1038/s41467-019-10668-1 (PMC6597584; doi:10.1038/s41467-019-10668-1)
Supplement: Supplementary file 1 — Supplementary Information [file 41467_2019_10668_MOESM1_ESM.pdf]

SUPPLEMENTARY INFORMATION

MorphoNet: An interactive online morphological browser to explore complex multi-scale data

Leggio et al.

Supplementary Figures.

We provide 10 Supplementary Movies illustrating the broad range of applicability of MorphoNet as morphological browser and giving example of utilization of several MorphoNet features to explore the structure and dynamics of datasets.

An overview of these movies is given in Supplementary Figure 1.

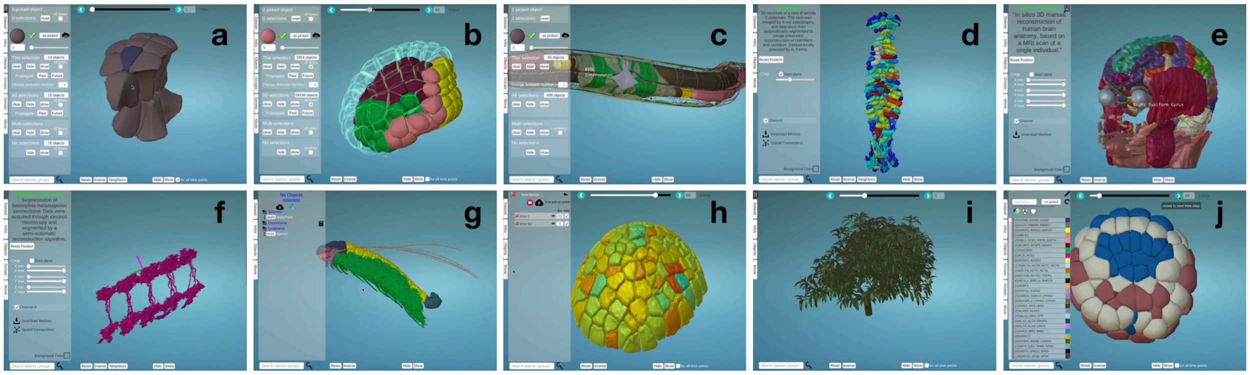

Supplementary Figure 1. MorphoNet features and video tutorials. Screen captures of the ten Supplementary Movies. **a** Development of a *Phallusia mammillata* embryo, in which adjacency relationships between cells are explored. **b** Development of a *Phallusia mammillata* embryo, in which tissues have been color-coded. **c** Structure of the worm C. elegans, in which organs have been color-coded and parts of the body hidden from view in order to reveal internal structures. **d** Structure of the nest of *Cubitermes* termites. The heat map shows chambers volume distribution (red=high, blue=low). **e** Structure of a human head. Colors show different anatomical parts. **F** *Drosophila* medulla connectome, in which synapses have been color-coded according to their specific role and part of the structure is hidden from view, to reveal specific neural connections. **g** Body structure of *Cascolus ravitis* gen. et sp. nov., a fossil crustacean. Body parts are color-coded. **h** Development of a Zebrafish embryo. The heat map shows for each cell, the time until its next division (red=high, blue=low). **i** Simulated mango tree structure and growth. **j** Gene expression patterns during the embryonic development of *Phallusia mammillata*.

Supplementary Notes.

Several datasets are to this day available to public MorphoNet users, and several others are hosted on the MorphoNet.org server in private form.

We detail here the public and private datasets shown in the paper.

Datasets shown in the paper (in bold the dataset name on MorphoNet):

- *Phallusia mammillata* embryo. ***Phallusia mammillata* (Wild type, live SPIM imaging, stages 8-17)** (**Figs. 1, 2, 3, Supplementary Fig. 1, Supplementary Movies 1, 2, 10**): live embryonic development of Phallusia mammillata (Phlebobranch ascidian). Cell membranes were fluorescently labeled by microinjection of mRNA encoding PH-GFP into unfertilized egg. The embryo has been live imaged with a MuVi-SPIM[1] for six hours, from the 64-cell stage (Tp 1, St. 8) up to the initial tailbud (Tp 180, St. 17). Whole cells were segmented using the ASTEC pipeline[2]. Surface meshes were produced using the VTK library and MeshLab. Public MorphoNet dataset.

- Human body structure. **Human body structure (Healthy adult, full-body MRI)** (**Figs. 1, 2**): *in silico* reconstruction of human body anatomy. This 3D model of human anatomy has been extracted from full-body MRI images and then manually curated and integrated using a 3D editing program. Anatomical structures are represented by 3D surface data, in which objects represent segments of

a three-dimensional whole-body model of an adult human male. Data available online at https://dbarchive.biosciencedbc.jp/en/bodyparts3d/desc.html. Public MorphoNet dataset.

- Simulated shoot-apical meristem. **Shoot-apical meristem (Simulated, oryzalin-treated organ formation)** (**Fig. 2**): Simulation of an abstract oryzalin-treated organ, growing out of a meristem filled with uniform and steady turgor pressure. The outgrowth is obtained by locally softening the cell wall. The growth algorithm is detailed in Boudon-2015[3] and the isotropic wall elasticity is described according to the model detailed in Oliveri-2018[4]. Data kindly provided by H. Oliveri. Public MorphoNet dataset.

- Termite nest. **Nest of termites *Cubitermes* (X-ray tomography)** (**Fig. 2, Supplementary Fig. 1, Supplementary Movie 4**): 3D reconstruction of a nest of termites Cubitermes. The nest was imaged using X-ray tomography with a medical scanner and reconstructed into a series of virtual cuts. Data were then automatically reconstructed with a dedicated reconstruction software[5]. Dataset kindly provided by A. Perna. Public MorphoNet dataset.

- *Caenorhabditis elegans* worm. ***Caenorhabditis elegans* (Wild type, adult)** (**Fig. 2, Supplementary Fig. 1, Supplementary Movie 3**): *in-silico* reconstruction of the adult worm *C. elegans*. This 3D model of worm anatomy has been created in Blender, based on biological data and information taken from WormAtlas (http://www.wormatlas.org) and Worm Image repository (http://www.wormimage.org). Data available online at http://caltech.wormbase.org/virtualworm/, constituting the primary dataset of the online platform OpenWorm (http://openworm.org). Public MorphoNet dataset.

- *Drosophila melanogaster* connectome. ***Drosophila melanogaster* medulla connectome (Wild type, TEM imaging, adult)** (**Fig. 2, Supplementary Fig. 1, Supplementary Movie 6**): segmentation of *Drosophila melanogaster* synaptic circuits of seven columns in the second neuropil or medulla behind the fly's compound eye. Data were acquired by electron microscopy and segmented using a semi-automatic reconstruction algorithm[6]. Public dataset, available at https://www.janelia.org/project-team/flyem/data-and-software-release. Public MorphoNet dataset.

- *Arabidopsis thaliana* meristem (**Fig. 3**). It consists of the meristem of *A. thaliana*, in which cell membranes have been fluorescently marked. The meristem has been imaged for several days. Microscope images have then been segmented, and surface meshes have been produced using the VTK library. Unpublished data, kindly provided by J. Traas. Private MorphoNet dataset.

- Human brain structure. **Atlas of human brain (Healthy adult, MRI)** (**Supplementary Fig. 1, Supplementary Movie 5**): *in silico* reconstruction of human brain anatomy. Images were captured by MRI and then manually reconstructed. Data available online at https://www.spl.harvard.edu/pages/Software. Public MorphoNet dataset.

- Crustacean fossil. ***Cascolus ravitis* (Fossil, digitally captured images)** (**Supplementary Fig. 1, Supplementary Movie 7**): 3D reconstruction of the fossil crustacean Cascolus ravitis gen. et sp. nov.[7] Data were collected by fossil grinding and image capture at 20 micron intervals. Volume reconstruction and segmentation was performed using the custom SPIERS software[8]. Data available online at https://datadryad.org/resource/doi:10.5061/dryad.g1q8p. Public MorphoNet dataset.

- Zebrafish embryo. **Danio rerio embryo (Wild type, live SGH 2-photon imaging, 1- to 510-cell stages)** (**Supplementary Fig. 1, Supplementary Movie 8**): live embryonic development of the Zebrafish (Danio rerio). Images were collected by Second Harmonic Generation microscopy of unlabeled embryos from the 1-cell to the 510-cell stage. Whole cell segmentation was performed by the automated segmentation algorithm of the BioEmergences workflow[9]. Data available online at http://bioemergences.iscpif.fr/Science2010/datasets.php. Public MorphoNet dataset.

- Mango tree structure. **Mango tree (Simulated, growth)** (**Fig. 1, Supplementary Fig. 1, Supplementary Movie 9**): *in-silico* simulation of Mango tree structure and growth. Unpublished data, kindly provided by F. Boudon. Public MorphoNet dataset.

Supplementary References.

1. Krzic, U., Gunther, S., Saunders, T. E., Streichan, S. J. & Hufnagel, L. Multiview light-sheet microscope for rapid in toto imaging. *Nat. Methods* **9**, 730–733 (2012).
2. Guignard, L. *et al.* Contact-dependent cell communications drive morphological invariance during ascidian embryogenesis. (2017). doi:10.1101/238741
3. Boudon, F. *et al.* A computational framework for 3D mechanical modeling of plant morphogenesis with cellular resolution. *PLoS Comput. Biol.* **11**, e1003950 (2015).
4. Oliveri, H., Traas, J., Godin, C. & Ali, O. Regulation of plant cell wall stiffness by mechanical stress: a mesoscale physical model. *J. Math. Biol.* (2018).
5. Perna, A. *et al.* The structure of gallery networks in the nests of termite Cubitermes spp. revealed by X-ray tomography. *Naturwissenschaften* **95**, 877–884 (2008).
6. Takemura, S.-Y. *et al.* Synaptic circuits and their variations within different columns in the visual system of Drosophila. *Proc. Natl. Acad. Sci. U. S. A.* **112**, 13711–13716 (2015).
7. Siveter, D. J., Briggs, D. E. G., Siveter, D. J., Sutton, M. D. & Legg, D. A new crustacean from the Herefordshire (Silurian) Lagerstätte, UK, and its significance in malacostracan evolution. *Proc. Biol.*

Sci. **284**, (2017).

8. Sutton, M. D., Garwood, R. J., Siveter, D. J. & Siveter, D. J. Virtual palaeontology toolkit. Available at: https://palaeo-electronica.org/content/issue-2-2012-technical-articles/226-virtual-palaeontology-toolkit. (Accessed: 27th July 2018)
9. Olivier, N. *et al.* Cell lineage reconstruction of early zebrafish embryos using label-free nonlinear microscopy. *Science* **329**, 967–971 (2010).
