# Supplementary material for: MorphoNet: an interactive online morphological browser to explore complex multi-scale data
Source: Nat Commun. 2019 Jun 27;10:2812. doi: 10.1038/s41467-019-10668-1 (PMC6597584; doi:10.1038/s41467-019-10668-1)
Supplement: Supplementary file 3 — Description of Additional Supplementary Files [file 41467_2019_10668_MOESM3_ESM.pdf]

Description of Additional Supplementary Files

File name: Supplementary Movie 1

Description: Cell adjacency exploration in a *Phallusia mammillata* embryo. First neighbours of a selected cells are picked through the Neighbors button and then color-labeled. The rest of the embryo is then hidden from view to visualize the full set of neighboring cells. Second-order neighbors of a different cell at a later developmental stage are then selected by using twice the Neighbors function.

File name: Supplementary Movie 2

Description: Development of a *Phallusia mammillata* embryo, in which tissues have been color-coded. Tissues are selected through the Groups menu and, to each of them, a color selection is applied through the Objects menu. The development is then explored in time by using the time scroll-bar, indicating the corresponding developmental stage in hours-post-fertilisation (hpf). Epidermis is also removed from view by hiding the corresponding selection from the Objects menu, and internal cells are visualised by cropping the dataset from the Dataset menu

File name: Supplementary Movie 3

Description: Structure of the worm *C. elegans*, in which organs have been color-coded and parts of the body hidden from view in order to reveal internal structures. Specific organs are selected from the Groups menu while the rest is hidden from view. Previously saved selections, labelling each anatomical group, are then applied from the Infos menu to show inner organs distribution. Previously saved names, corresponding to each organ, are then activated and shown as the mouse pointer passes over each object in the dataset. The whole worm anatomy is then shown by hiding its hypodermis.

File name: Supplementary Movie 4

Description: Structure of the nest of *Cubitermes* termites. The outer layer is hidden to reveal the inner structure, made of chambers and corridors. A heat map is then applied to represent the volume of each chamber. Finally the dataset is cropped along different fixed directions to reveal the connections between chambers.

File name: Supplementary Movie 5

Description: Structure of a human head, with colors marking different anatomical parts. The skin is hidden from view to reveal internal structures. Previously saved colors in the Infos menu are then applied to each object , identifying anatomical structures. Names labelling these structures are also shown as the pointer passes over each object. Inner features are explored by cropping the datasets to reveal internal organs.

File name: Supplementary Movie 6

Description: *Drosophila* medulla connectome, in which synapses have been color-coded according to their specific role and part of the structure is hidden from view, to reveal specific neuronal connections. Previously saved selections and names are applied from the Infos menu to label each type of synapses. The dataset is then cropped to better explore its connections, and one particular type of synapses is finally singled out by hiding all other selections, to reveal the neuronal connections between two layers of synapses.

File name: Supplementary Movie 7

Description: Body structure of *Cascolus ravitis* gen. et sp. nov., a fossil crustacean. Previously saved selections are applied from the Infos menu, to label each part of the reconstructed body.

File name: Supplementary Movie 8

Description: Development of a Zebrafish embryo. A heat map is applied from the Infos menu to each object, quantifying the time spent by each cell before division. A movie is then made from the Movie menu, showing the embryonic development and the time evolution of the heat map

File name: Supplementary Movie 9

Description: Simulated mango tree structure and growth. A color map is applied from the Infos menu to label leaves and branches. The tree growth is then explored through the timebar.

File name: Supplementary Movie 10

Description: Gene expression patterns during the development of *Phallusia mammillata* embryo. Genes are selected from the Genetic menu. The first gene (KH.C2.994) is selected among the ones expressed by a specific cell through the button *on picked*. The second gene (BPTF) is selected through the *search gene* option. Each expression territory is assigned a specific selection, whose number can be changed from the gene submenu. Through the time scrollbar, the expression dynamics of these two genes is explored. Cells expressing both genes at the same time show a blinking color selection. The overlap and the union of the two gene expressions is then shown by the overlap button
